# Influence of Loading History and Soil Type on the Normal Contact Behavior of Natural Sand Grain-Elastomer Composite Interfaces

**DOI:** 10.3390/polym13111830

**Published:** 2021-06-01

**Authors:** Yu Tian, Sathwik S. Kasyap, Kostas Senetakis

**Affiliations:** Department of Architecture and Civil Engineering, City University of Hong Kong, Kowloon Tong, Hong Kong, China; ytian52-c@my.cityu.edu.hk (Y.T.); ssarvadev2-c@my.cityu.edu.hk (S.S.K.)

**Keywords:** recycled rubber, elastomer, contact mechanics, earth material, composite interface

## Abstract

Recycled rubber in granulated form is a promising geosynthetic material to be used in geotechnical/geo-environmental engineering and infrastructure projects, and it is typically mixed with natural soils/aggregates. However, the complex interactions of grains between geological materials (considered as rigid bodies) and granulated rubber (considered as soft bodies) have not been investigated systematically. These interactions are expected to have a significant influence on the bulk strength, deformation characteristics, and stiffness of binary materials. In the present study, micromechanical-based experiments are performed applying cyclic loading tests investigating the normal contact behavior of rigid–soft interfaces. Three different geological materials were used as “rigid” grains, which have different origins and surface textures. Granulated rubber was used as a “soft” grain simulant; this material has viscoelastic behavior and consists of waste automobile tires. Ten cycles of loading–unloading were applied without and with preloading (i.e., applying a greater normal load in the first cycle compared with the consecutive cycles). The data analysis showed that the composite sand–rubber interfaces had significantly reduced plastic displacements, and their behavior was more homogenized compared with that of the pure sand grain contacts. For pure sand grain contacts, their behavior was heavily dependent on the surface roughness and the presence of natural coating, leading, especially for weathered grains, to very high plastic energy fractions and significant plastic displacements. The behavior of the rigid–soft interfaces was dominated by the rubber grain, and the results showed significant differences in terms of elastic and plastic fractions of displacement and dissipated energy compared with those of rigid interfaces. Additional analysis was performed quantifying the normal contact stiffness, and the Hertz model was implemented in some of the rigid and rigid–soft interfaces.

## 1. Introduction

Recycled rubber in a granulated or shredded form derived from waste automobile tires has been applied in geotechnical and geo-environmental engineering projects over the last three decades, both in pure form and mixed with earth materials. Recycled rubber is an elastomer type of polymer with very low specific gravity and high energy dissipation properties; thus, it has very attractive properties to be used in a variety of applications such as lightweight geosynthetic, alternative and low-cost vibration isolation earth material, or drainage earthen system in landfills [1,2,3,4,5,6,7,8,9,10]. Many research studies have proposed the use of recycled rubber in granulated/shredded form in various projects, for example, as lightweight embankment/subgrade material [11,12,13,14,15,16], backfill in retaining walls [12,17,18,19,20], high damping capacity system beneath foundations [21,22,23,24], isolation material mitigating soil liquefaction [25,26,27,28], and railway ballast [29]. Recycled rubber, apart from being used in geotechnical engineering as a lightweight material, also finds a variety of other potential applications such as composite material in new concrete production or asphalts [30,31,32], and it may find applications in industrial and aeronautic engineering as well [33,34,35].

In geotechnical engineering, a significant amount of research has been conducted investigating the mechanical and dynamic properties of granulated rubber and soil–rubber mixtures [7,28,36,37,38,39,40,41,42,43,44,45,46,47]. However, because of the complex nature of soil–grain–rubber interactions, which heavily control the bulk behavior of these granular composites, there are still many unresearched areas, especially in the analysis of these binary materials at the micromechanical level and multi-scales [46,48,49,50]. Similar to the behavior of other binary materials composed of rigid and soft grains, for example, sand–expanded polystyrene beads (sand–EPS) [51,52,53], the investigation into the (bulk) constitutive behavior of sand–rubber mixtures necessitates an understanding of the way viscoelastic (soft) grains interact with brittle-to-ductile (rigid) grains, which can help researchers to develop multi-scale models in the analysis of these granular composites. As there exists a gap in the literature by means of fundamental studies that look into the problem of rigid–soft grain interaction at the small-scale based on laboratory observations, the present work attempted to provide a new contribution in this direction.

In their recent experimental study based on grain-scale and element-size samples of sand–rubber mixtures, Li et al. [46] reported a direct relationship between the grain-to-grain friction (interparticle coefficient of friction) with their bulk strength. Previous experimental and theoretical studies reported the important influence of both rubber content and the relative size of sand against rubber particles on the bulk behavior of these composites in terms of strength, compression/deformation behavior, and stiffness [40,41,54,55,56,57]. These influences have been linked predominantly to the distribution of contacts between sand–sand, sand–rubber, and rubber–rubber grains, influencing the dynamic development of load transfer networks within the granular assembly.

The application of granulated/shredded rubber in ground improvement has also been proven beneficial in cases that the host soil is composed of crushable/weak grains. An example of this may refer to the application of chemically decomposed rocks such as completely weathered granite or volcanic materials, which are abundant in tropical–subtropical regions, and they are commonly used as earthen materials in various projects. However, these materials are characterized by high compressibility, which is contributed by the low strength of the individual grains; the low particle strength also contributes to the amplification of creep deformations in those materials. The studies by Fu et al. [43,44,58] and Li et al. [47] showed that the inclusion of granulated rubber provides a mitigating mechanism against sand grain breakage, even though, because of the deformable nature of the polymeric grains, the inclusion of granulated/shredded rubber may increase the compressibility of the binary material. This behavior may depend upon the content of rubber as well as the relative size of sand and rubber particles. In their recent study, Tian and Senetakis [59] showed that the inclusion of granulated rubber in sands might have a beneficial influence in increasing bulk stiffness as observed in crushable sand–rubber mixtures while providing a mechanism of mitigating creep deformations due to the reduction of sand grain breakage. However, Tian and Senetakis [59] noticed that the overall mechanisms contributed by the inclusion of rubber are complex, as the presence of this polymeric material in granulated form provides, simultaneously, different and competitive mechanisms in soil creep. This complex behavior is ascribed to the viscoelastic nature of rubber grains contributing to the reduction of contact stresses within the granular assembly, thus preventing sand grain breakage, and by deforming continuously with time, altering in this way the deformation characteristics of binary materials. Indeed, this behavior may lead to a significant increase in the stiffness of sand–rubber mixtures within the service period of geo-systems where granulated rubber is used as geosynthetic.

Based on the abovementioned complexities, as revealed in previous works on sand–rubber mixtures, it is essential that future studies attempt to investigate the behavior of these binary materials through micromechanical-based simulations, for example, using the discrete element method (DEM), which is a powerful tool in understanding the small-scale fundamental mechanisms that control the behavior of earthen and binary (or composite) materials. DEM studies use as input the interparticle friction and contact laws of the interacting grains, and this input has been proven to have a significant influence on the resultant output (or bulk behavior) of granular materials [60,61,62]. Thus, understanding and modeling the contact behavior of composite sand–rubber interfaces is essential to be obtained through grain-scale experimental studies, which was one of the major motivations behind this work. Even though previous studies using DEM attempted to provide insights into the fundamental mechanisms that control the mechanical behavior of binary systems composed of rigid and soft grains, including sand–rubber [56,57,63,64,65,66,67], as well as sand–expanded polystyrene beads [51,68], there has been reported much less progress in terms of laboratory studies at the grain-scale [46,49]. Especially the works by Li et al. [46] and He et al. [49] had a major focus on the interparticle coefficient of friction and the constitutive behavior in the shearing (or tangential) direction of sand–rubber composite interfaces. However, a complete understanding of the fundamental behavior of interfaces necessitates an investigation of the normal load–displacement response and normal contact stiffness variation, which are important to examine by means of cyclic tests. This investigation is important because, in natural systems, the contact force networks alter dynamically; thus, grain contacts are continuously subjected to changes of the magnitude of applied normal load.

In the present study, an experimental investigation into the normal contact behavior of sand–rubber composite interfaces is attempted examining the influence of sand particle type, number of loading cycles, and preloading by performing micromechanical-based (grain-scale) experiments. Emphasis is placed on the analysis and quantification of the elastic and plastic fractions of displacement, the energy dissipation, and the understanding of the contributing mechanisms of the rubber particles due to their viscoelastic nature, as well as the analysis of the data by means of a contact model commonly used in the study of non-conforming surfaces. This analysis contributes to a better qualitative understanding, at the grain-scale, of the behavior of composite materials composed of rigid (sand) and soft (rubber) particles and can also provide input parameters to be used in DEM analyses of binary granular systems and geosynthetics. Thus, the main objectives of this work are summarized as follows: (i) examining the influence of natural grain type and its morphology on the response of sand–rubber composite interfaces covering a range of natural materials (in sand–rubber systems); (ii) providing a qualitative and quantitative understanding of the influence of the number of loading cycles and pre-loading on the normal contact behavior of sand–rubber composite interfaces; (iii) analyzing energy dissipation mechanisms and the role of loading history; (iv) analytically studying the problem of the normal contact response of sand–rubber interfaces based on the laboratory test results. Based on the existing literature and abovementioned major gaps, this work comprises a new contribution into the systematic analysis and quantification of the normal contact behavior of sand–rubber interfaces considering a large number of potential influencing factors, which can provide a direct contribution of input parameters in DEM simulations of binary granular materials. 

## 2. Materials and Methods

### 2.1. Materials Used and Their Compositional and Morphological Characterization

Three types of natural aggregates (sands) from different geological origins (2–5 mm fractions) and a polymeric material in granulated form composed of shredded automobile tires were used in the micromechanical-based experiments. The three natural aggregates (LBS, BLS, CDG) have different morphological features both at the scale of the grain size and also at the small-scale of roughness. Leighton Buzzard sand (denoted as LBS) is a natural quartz sand from the UK, which consists of subrounded to rounded grains of relatively smooth texture. Blue sand (denoted as BLS) is a silica-based material from New South Wales, Australia, and it is composed of crushed rock of mafic magma with highly angular grains and rough texture. Completely decomposed granite (denoted as CDG) originates from Hong Kong, and it consists of a weathered igneous rock of felsic composition, and its grains are crushable (i.e., weak grains) with irregular shapes and rough texture. LBS is a benchmark quartz sand previously studied in the Geomechanics Laboratory of City University of Hong Kong [69,70,71], BLS is a typical material (in crushed form) used as fill/backfill/ballast in infrastructure projects [72], while CDG may also find many potential applications as an earth material in tropical/subtropical regions such as Hong Kong [47,73,74,75].

**Figure 1:**
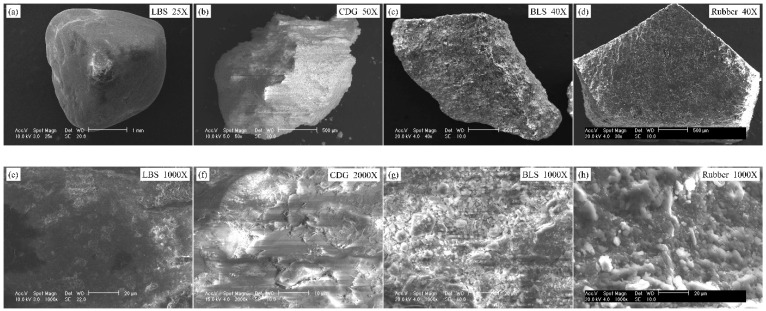
SEM images of sand grains and granulated rubber (**a**–**d**) at low magnification and (**e**–**h**) at high magnification.

**Figure 2:**
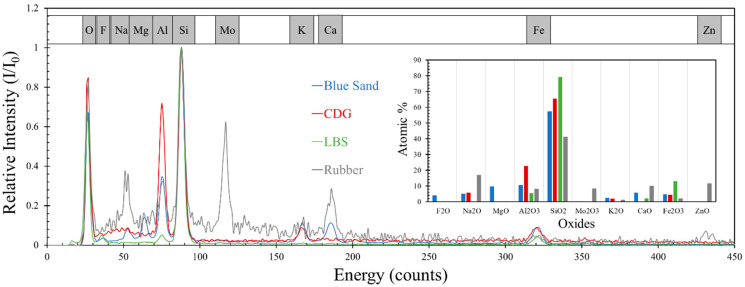
Element spectra of four materials tested and atomic percentages of corresponding oxides in each material.

Scanning electron microscope (SEM; FEI/Philips XL30 Esem-FEG, FEI Company, Hillsboro, OR, USA) images of representative grains from the different samples (natural and polymeric grains) at different magnifications are given in Figure 1. The high magnification SEM images of these samples showed highly distinctive surface conditions. LBS grains consist of relatively homogeneous surface textures, whereas BLS and CDG samples have adsorption of debris (microparticles) on their surfaces (more prominently for CDG), which consist, primarily, of clay particles on a dominant matrix of quartz (indicated in Figure 1f,g). The origin of this debris is, primarily, because of the mining process (for BLS) and aggressive chemical weathering (for CDG) subjected on the original rocks. The compositional characterization of the materials was performed based on energy dispersive spectroscopy (EDS) analysis, carried out along with SEM, and representative results are displayed in Figure 2. Based on this analysis, the three natural aggregates have quartz as the dominant mineral taking up 60–80% of SiO_2_ formations. The LBS grains have minor traces of impurities from iron-oxides, while the CDG grains have, apart from the dominance of quartz, significant quantities of aluminum-oxides (23%), which can be associated with the clay minerals (along with Na and K elements), resulted from the chemical weathering of feldspars and micas from the original igneous rock. BLS grains have formations of lightweight alkali earth metal oxides (Mg, Na, and Ca), most presumably due to the mining of the original basaltic rock. The presence of debris materials on the grain surfaces changes the micro-scale morphology of the aggregates and is expected to have a significant influence on their inter-particle contact behavior, as previous studies would also suggest [73,76,77,78].

The polymeric grains included in the study to investigate the behavior of composite interfaces can be classified as granulated rubber (with an average size similar to that of the sand grains), based on the ASTM specifications [10]. The elemental composition analysis of this sample showed significant amounts of Molybdenum (Mo), which forms the reinforcing mechanism in natural rubber. The rubber particles are obtained from recycled automobile tires that have been shredded, forming granules of various sizes and shapes. In the present study, polymeric grains with relatively flat surfaces were chosen for the experiments of rigid–soft interfaces (rigid: soil grain, soft: rubber grain). Inspection of SEM images of representative granulated rubber samples revealed the presence of some surface cracks on the material and that the polymeric grains have a highly continuous and uniform texture.

The SEM images in Figure 1 can provide some qualitative inferences on the surface texture at the meso-scale of the different types of aggregates, whose textural characteristics may have an important influence on their tribological behavior, for example, the presence of debris [78,79] or meso-scale morphology [80]. However, the surface roughness at the micro-scale (expressed with an RMS value in the present study) is itself an important characteristic that controls the frictional and constitutive behavior of interfaces [70,81,82] and also the bulk behavior of granular materials [46,60,61,62,83,84,85]. For quantitative evaluation of surface roughness, an optical surface profiler (Wyko NT9100 Surface Profiler, Veeco Instruments Inc., Tucson, AZ, USA) was used, and the surface roughness values were calculated from the standard deviation of the asperity peaks from the mean height (*S_q_* as shown in Equation (1)) for a given area.

(1) $$S_{q}=\sqrt{\frac{1}{u}\overset{u}{\underset{i=1}{\sum }}\left(w_{i}{}^{2}\right)}$$

where *u* is the number of measured data points and *w* is the elevation relative to the base surface. 

**Figure 3:**
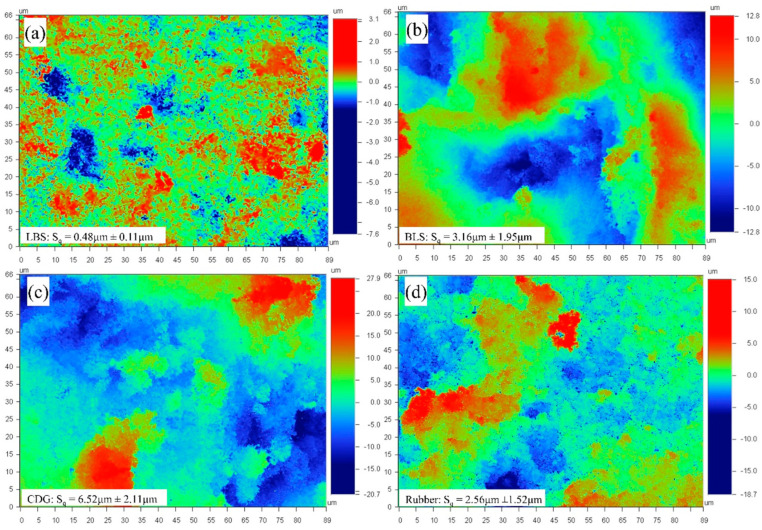
2D surface profiles with scanned area of 66.4 × 88.7 μm of (**a**) LBS, (**b**) blue sand, (**c**) CDG, and (**d**) rubber.

**Figure 4:**
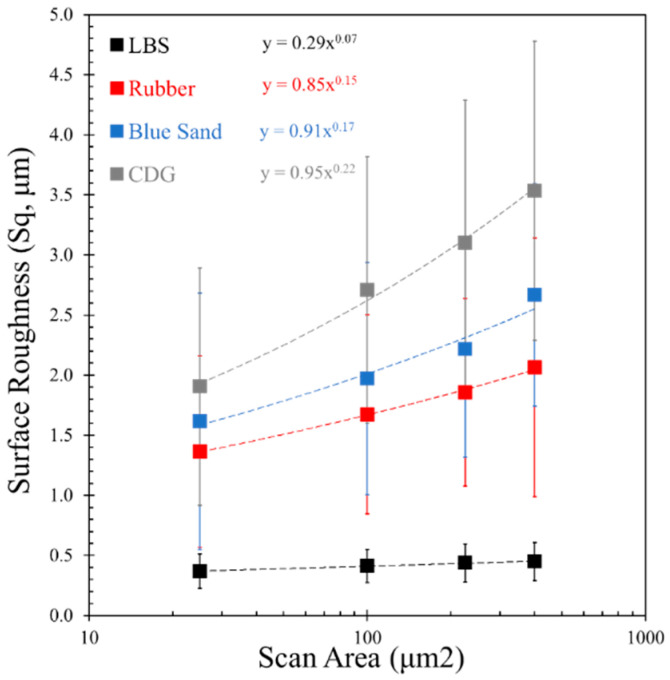
Variation of surface roughness values with different scanned areas.

Representative flattened surface profiles of the four types of aggregates for a given scanned area of 67 μm × 89 μm are shown in Figure 3, and the average *S_q_* values along with one standard deviation from 10 different measurements for each material type are displayed in Figure 4. It was observed that the average *S_q_* value for a given material increased with a higher scanned area, and the trend of increment in *S_q_* was different for each material type. A systematic calculation of *S_q_* values for scanned areas ranging from 25 to 400 μm^2^ was performed with a constant data resolution of 0.39 Mdpi (or 240 datapoints per 1 μm^2^).

The data in Figure 4 suggest that for the LBS grains, which displayed the smoothest surfaces (i.e., the lowest values of *S_q_*), the RMS roughness is almost independent on the scanned areas (power coefficient of 0.07). Granulated rubber and BLS particles have an intermediate variation (power coefficients of 0.15 and 0.17, respectively) of surface roughness with scanned area owing to their asperities formed from their respective origins. CDG grains, because of the non-uniform clay coating on their surfaces and their generally very rough textures, show significantly higher surface roughness values and also a faster increase in *S_q_* values with the scanned area (power coefficient of 0.22). It can be inferred that the surfaces with higher average *S_q_* values for a given area show greater dynamics in the lateral variation of asperity heights and that the values of the standard deviation of the surface roughness for different locations for a given material type are significantly high. This analysis is particularly important in the present study, because the contact area between sand and rubber particles is expected to significantly increase during the application of normal loading, owing to the viscoelastic behavior of the granulated rubber. For pure sand grain contacts, even though some increase of the apparent area, as a result of normal load increase, is expected based on Hertz contact response (after [86]), such an increase would be considered very small compared to that of rigid–soft contacts.

### 2.2. Experimental Setup for Normal Contact Tests

**Figure 5:**
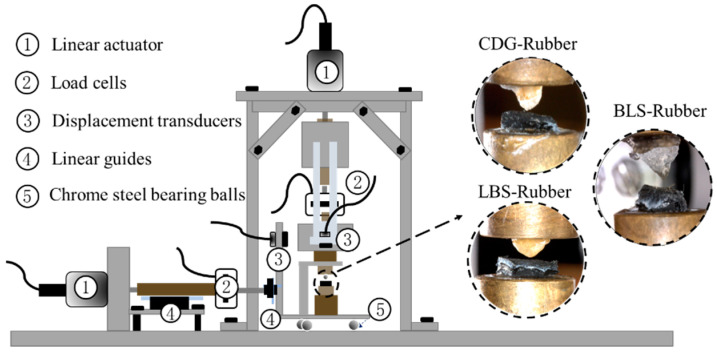
Schematic diagram of the testing apparatus and the testing materials.

The grain-scale (interface) experiments were performed using a custom-built micromechanical apparatus at City University of Hong Kong, which is capable of testing sand-sized grains ranging, approximately between 0.5 and 5.0 mm, with grains having apex–apex, apex–block, or block–block types of contacts. Previous works using the same apparatus have studied, predominantly, pure sand grain contacts as well as block types of contacts typically of the “rigid-type” [69,70,71,75,81,82,87,88,89] and have provided detailed technical descriptions of the apparatus and its calibrations. A schematic illustration of the experimental setup is given in Figure 5.

The apparatus consists of three loading arms/systems and has the capability to apply/recode forces and displacements both horizontally (i.e., in the shearing and out-of-plane directions) and vertically (normal to the shearing plane). Each of the three loading systems of the apparatus consists of a set of stiff mechanical parts, linear bearings, a linear actuator (Zaber Technologies, Vancouver, BC, Canada), a load cell (Novatech Measurements Ltd., East Sussex, England), and a non-contact displacement transducer (Microepsilon Messtechnik GmbH&Co., Ortenburg, Germany). Both the load cells and displacement sensors have very high precision, which helps to resolve the data for the derivation of contact stiffness (both in the shearing/tangential and normal directions). Each grain is fixed on a mount that is rigidly connected to the guiding sled (lower particle) and the vertical loading system (upper particle). During the application of the vertical (normal) loading, the lower grain is held stationary while the upper grain can move downwards and upwards under a displacement-controlled or force-controlled mode for the application of the loading and unloading phases, respectively.

### 2.3. Testing Program

**Figure 6:**
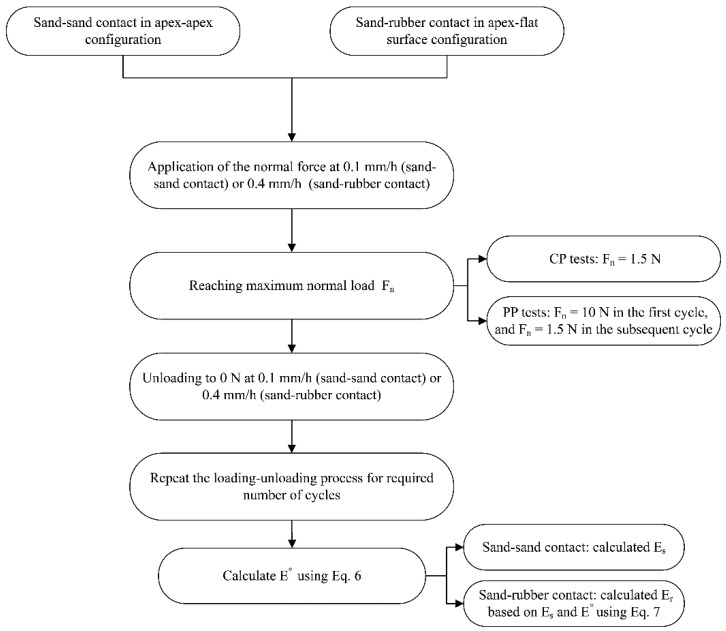
Flowchart explaining the different steps during the experiments and data analysis.

The micromechanical experiments in the present study on sand–sand (“rigid” systems) and sand–rubber grain interfaces (“rigid–soft” systems) involved two major classes: (i) cyclic normal loading tests investigating the influence of sand type and the number of loading cycles. These tests (termed “CP”) were performed applying 10 loading–unloading cycles up to a maximum normal load of 1.5 N and (ii) preloading path tests investigating the influence of loading history (or preloading) on the behavior of the grain contacts. For this class (termed as “PP”), the first cycle was applied at a maximum normal load of 10 N and was followed by 9 cycles at a maximum normal load of 1.5 N. Thus, the influence of previous loading history in terms of a greater applied load could be investigated at the contacts of aggregate-rubber. The experiments were performed at a loading rate of 0.1 mm/h for rigid interfaces and 0.4 mm/h for rigid–soft interfaces for both the loading and unloading phases. Before the first cycle of loading, the contact between the top and bottom grains was first ensured after the application of a seating load of around 10 mN. A summary of the experiments and the combinations of grain interfaces is given in Table 1. A flowchart explaining the experimental process and respected analysis from the grain-scale tests (also linking the different steps with the subsequent analytical expressions) is given in Figure 6.

## 3. Results and Discussion

### 3.1. Cyclic Normal Load Tests without Preloading (CP Tests)

#### 3.1.1. Rigid Interfaces

**Figure 7:**
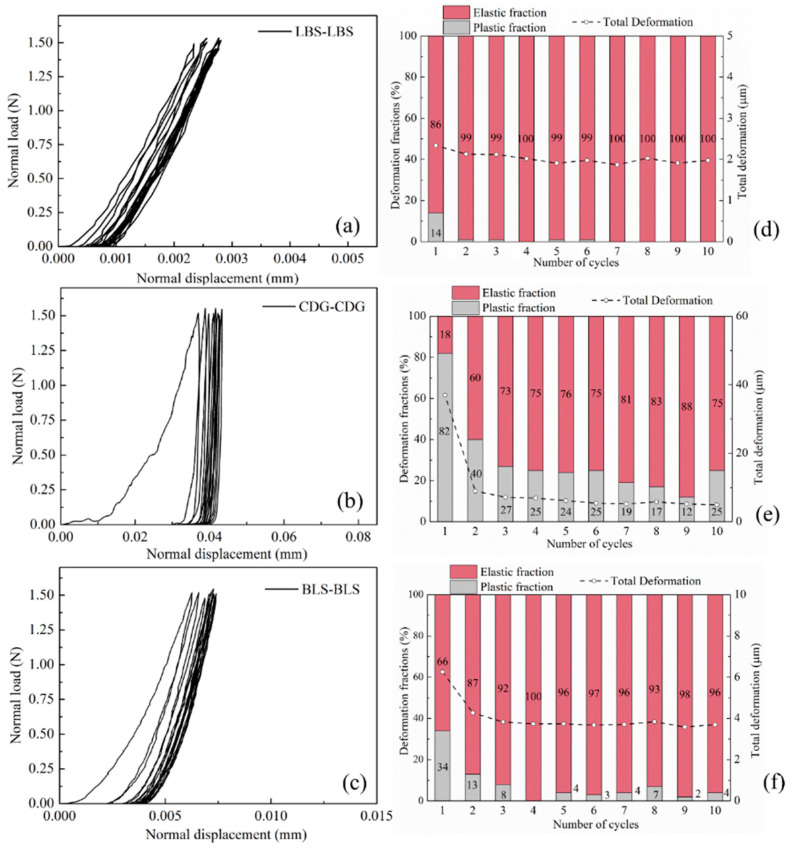
Load–displacement curves of cyclic loading for LBS–LBS, CDG–CDG, and BLS–BLS (**a**–**c**) and corresponding displacement fractions (**d**–**f**).

In the following discussions, the total deformation of the specimens (or grain system) is defined as the displacement measured at the maximum normal load, and the elastic and plastic fractions are the recovered and unrecovered parts, respectively, which are quantified upon uploading. The load–displacement response of the three types of rigid contacts (pure contacts of LBS–LBS, CDG–CDG, and BLS–BLS) are illustrated in Figure 7a–c. The variations of the total displacement (dashed line), and the corresponding elastic and plastic fractions within each loading cycle are plotted in Figure 7d–f. The results suggested that the LBS–LBS contacts display predominantly elastic response with 86% of elastic fraction and 2 μm total displacement in the first cycle, after which the subsequent cycles resulted in purely elastic behavior (Figure 7d). The virgin loading cycle of CDG–CDG contact displayed a softer response, reaching a total displacement of around 35 μm (~18 times higher than that of LBS–LBS contact) at the maximum normal load, and 82% of this displacement is irrecoverable upon unloading (Figure 7e). Despite the fluctuations in the data, the total displacement to reach 1.5 N normal load decreased with the increasing number of cycles, and the plastic fraction also reduced from ~80% to ~20%. These data are in qualitative agreement with previous studies investigating the cyclic normal load behavior of decomposed tuff grains [76], which are characterized by a heavy coating of clay microparticles formed by the chemical weathering of the parent rock.

Kasyap et al. [78] defined the fundamental difference between the normal contact response of clay- and silt-coated (artificially coated) LBS grains in terms of the trend of non-linearity in the load–displacement curves, given that both the grain classes show significant compression compared to the uncoated grains. It was demonstrated based on micromechanical experiments and microscopic image observations that grains coated with clay, because of the smaller size and softer behavior of the microparticles compared with silt coating, tend to show smoother normal load–displacement curves without any abrupt drop or fluctuations in the normal load (ascribed to particle rearrangement of the silt microparticles in the study by [78]). In the present study, the CDG grains with non-uniform natural clay coating on their surfaces showed an intermediate behavior between clay- and silt-coated sand grains with higher compression and slightly irregular load–displacement curves (no abrupt drop in the normal load for the given range of normal loads and displacements for the CP tests).

The normal contact response of BLS grains in Figure 7c is comparable to LBS (Figure 7a) but with greater compression for a given normal load (i.e., lower loading stiffness) owing to the higher surface roughness and thus significant asperity breakage (after [82]). For BLS samples, the change of elastic and plastic fractions of displacement with the increase in loading cycles is similar to the behavior of LBS grains. The mobilized displacement at 1.5 N normal load for BLS grains in the first loading cycle was around 6 μm, with a plastic fraction of 34%; however, both values of mobilized displacement and plastic fraction gradually stabilized after the third cycle, with values of approximately 4 μm and 5%, respectively, despite the small fluctuations in the data. These results suggest that for chemically weathered grains of igneous origin, which are characterized by rough surfaces with the presence of heavy coating of clay-type microparticles, significant plastic displacements were present even in cycle-4 to cycle-10; however, for crushed aggregates of BLS, the plastic displacements almost diminished from cycle-4 and beyond that. Even though both CDG and BLS grains displayed rough surfaces, and both types of aggregates have inclusions of microparticles on their surfaces, CDG is predominantly characterized by a heavy clay coating, whereas BLS is characterized, primarily, by rough surfaces due to the mining process the original rock has been subjected to. Thus, over the ten loading cycles in the CP tests, CDG interfaces were dominated by the compression of the microparticles leading to an accumulation of plastic deformations, whereas for BLS, the major part of the deformations, caused by the plastic response of asperities, took place during the very first cycles of loading, leading, in the consecutive cycles, to a behavior that was very similar to that of LBS.

It is noted that geological materials (and respected interfaces of particles) are expected to display a brittle to ductile behavior; thus, the contact response of rough interfaces may be influenced by both plastic deformations of asperities as the contact mechanics literature would suggest [82,90] and, perhaps, some brittle damage of micro-asperities [73,79,82]. For the given range of normal loads applied in the CP tests and based on the generally smooth shape of the normal load–displacement curves, it is expected that plastic behavior is the dominant mechanism (at the maximum normal load of 1.5 N), which may be contributed by both the compression of the coating (for CDG and BLS) and asperity deformation for the three different types of sands. For rigid interfaces, the three major contributing factors on their normal contact response can be summarized as (i) microscale morphology (roughness); (ii) mesoscale morphology represented by the local shape of the grains in the vicinity of their contacts; and (iii) the presence of impurities on the surfaces of the grains. All the natural grains tested in the present study (despite their differences in terms of morphology and the presence of natural coating in some types of aggregates) are, predominantly, silica-based materials; thus, surface chemistry is important in terms of composition (i.e., elemental analysis) of the grains, as this would be expected to influence surface hardness [70,72,75]. As will also be discussed in the subsequent sections, there is a significant difference between the natural aggregates (as brittle-to-ductile materials) with that of granulated rubber, which belongs to the group of elastomers, and its behavior is viscoelastic, which plays a dominant role in the behavior of the composite interfaces.

#### 3.1.2. Rigid–Soft Interfaces

**Figure 8:**
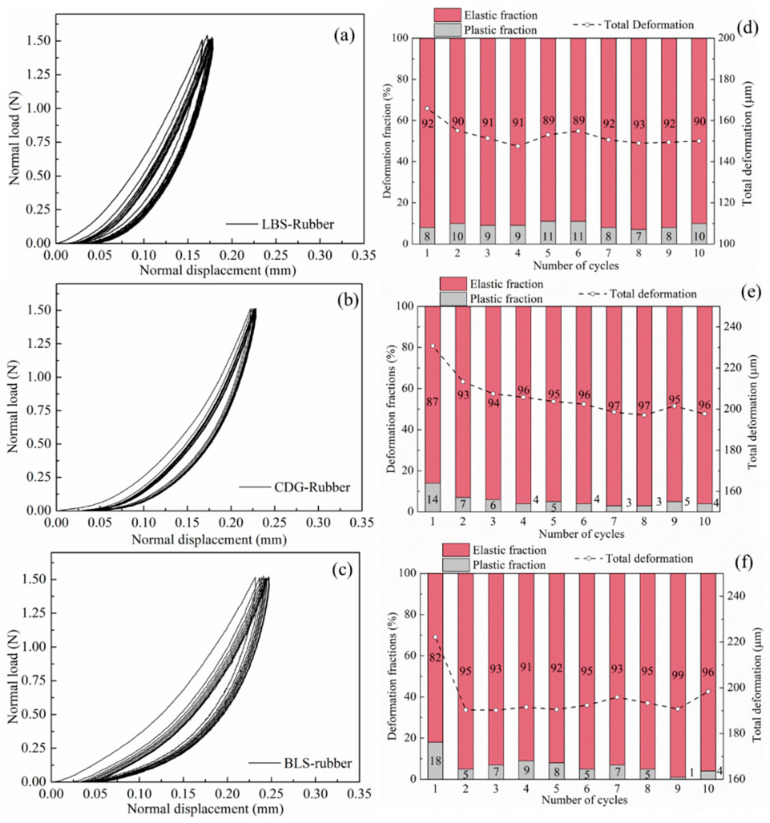
Load–displacement curves of cyclic loading for LBS–rubber, CDG–rubber, BLS–rubber (**a**–**c**) and coresponding displacement fractions (**d**–**f**).

The normal contact response of LBS–rubber, CDG–rubber, and BLS–rubber composite interfaces for ten loading cycles in CP tests is presented in Figure 8a–c, and the corresponding variations of elastic and plastic fractions at each cycle are compared in Figure 8d–f. The composite (rigid–soft) interfaces showed predominantly elastic behavior and significantly higher total displacements (on average 5 times for CDG, 30 times for BLS, and 64 times for LBS) compared to the pure sand grain contacts. However, the behavior of these composite interfaces cannot be considered purely elastic, as some small portion of plastic deformations was also observed at the end of each loading cycle. Though the fractions of plastic deformation decreased in the case of composite interfaces compared with pure sand grain contacts, their absolute values are significantly higher, particularly in cycle-1. For example, the LBS–LBS contact showed a plastic displacement of around 0.3 μm, corresponding to 14% plastic fraction with respect to the total displacement, whilst the LBS–rubber contact showed around 14 μm of plastic displacement, which was much larger compared to pure LBS interfaces; however, the plastic fraction for the composite interface (corresponding to 8% of the total displacement) was in general comparable with that of the pure LBS contact. For CDG–rubber and BLS–rubber interfaces, the portions of plastic displacement were significantly reduced in the first loading cycle compared with the respected results on pure CDG and BLS samples. In the subsequent cycles, all the types of composite interfaces showed a similar response in terms of plastic fractions of displacements, indicating a dominant influence of the rubber (viscoelastic in nature) particle.

**Figure 9:**
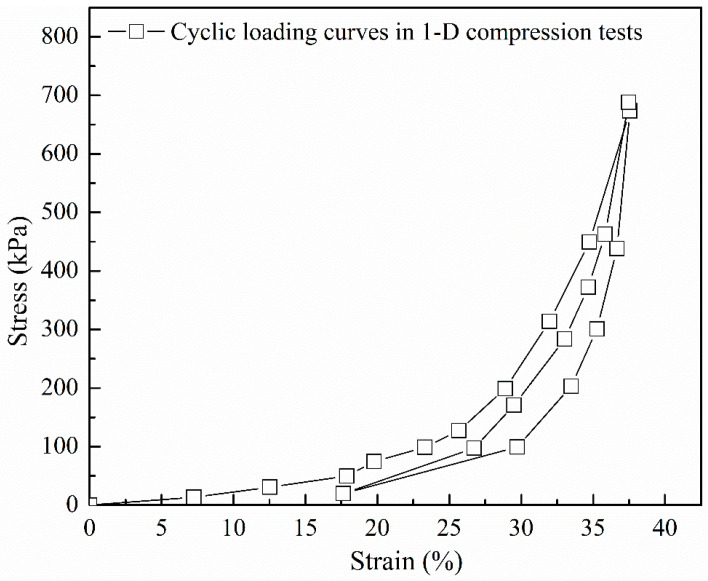
Displacement response for loading–unloading–reloading cycles in marcoscopic tests (reproduced by the authors, after Edil and Bosscher [2]).

From element-scale tests based on one-dimensional confined compression, Edil and Bosscher [2] reported large plastic strains for sand–rubber mixtures. The reproduced data by the authors from the Edil and Bosscher [2] study are displayed in Figure 9, and the stress–strain response (macroscopic behavior) showed, qualitatively, a great similarity with the interparticle compression tests (microscopic behavior). The studies by [2,91,92] ascribed such plastic responses to the rearrangement of the particles in the first cycle, assuming that the deformation of rubber is purely elastic. An additional mechanism was proposed by Valdes and Evans [63], in which study the observed large residual strains were also explained by the higher sidewall friction. The grain-scale experiments in the present study suggest that plastic deformations of the sand–rubber composite interfaces may also contribute, as an additional mechanism, to the bulk plastic behavior of binary mixtures, even though the inclusion of rubber prevents, to an important extent, large fractions of plastic displacement, especially for assemblies having crushable sand grains with irregular shapes or with rough textures.

**Figure 10:**
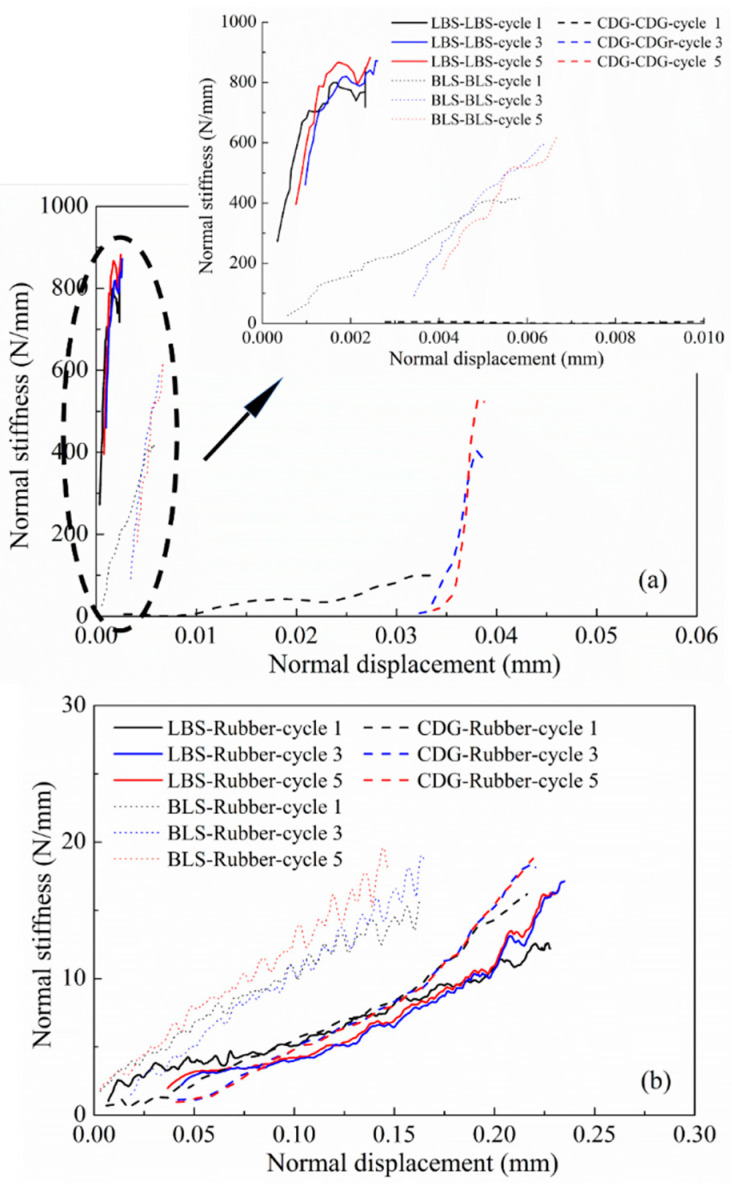
Normal contact stiffness of (**a**) rigid interfaces and (**b**) rigid–soft interfaces.

In DEM simulations of granular systems, the normal contact stiffness (*K_N_*) comprises one of the important input properties of the interacting grains. By differentiating the normal load against the displacement from the curves presented in Figure 7 and Figure 8, the normal contact stiffness against the displacement for the different grain combinations is presented in Figure 10 (data correspond to cycles 1, 3, and 5 during the loading process). For the pure sand grains, LBS had much larger K_N_ values compared to BLS and CDG, in a range of approximately 600–800 N/mm at normal displacements between about 1 and 2 μm (note the strong dependency of normal contact stiffness on the displacement in these curves). These values are, in general, four times greater compared with those of BLS contacts for the given displacement range. CDG grains displayed extremely small values of K_N_ during cycle-1; however, in subsequent loading cycles, the values were in general comparable with those of BLS. For the composite interfaces, K_N_ values were, on average, one to two orders of magnitude smaller compared with those of the pure sand grains, while the influence of the loading cycle was much smaller compared with that of the pure sand grain contacts (specifically CDG and BLS interfaces). Even though the presentation of these data (normal contact stiffness) attempts to provide some general understanding of a range of K_N_ values for the different material types, these results may also comprise some useful guide of input parameters in DEM simulations of binary materials.

### 3.2. Cyclic Normal Load Tests with Preloading (PP Tests)

#### 3.2.1. Rigid Interfaces

**Figure 11:**
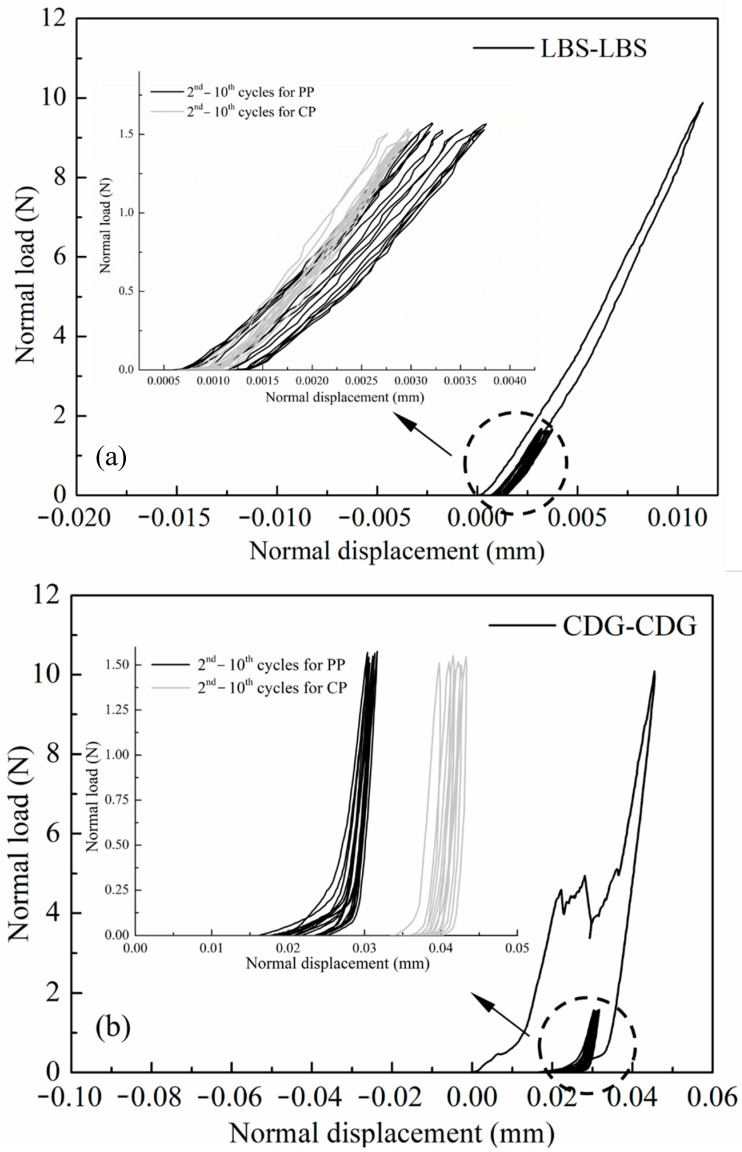
Load–displacement curves of preloading for (**a**) LBS–LBS and (**b**) CDG–CDG contacts and their comparisons with cyclic tests (inset Figures).

**Figure 12:**
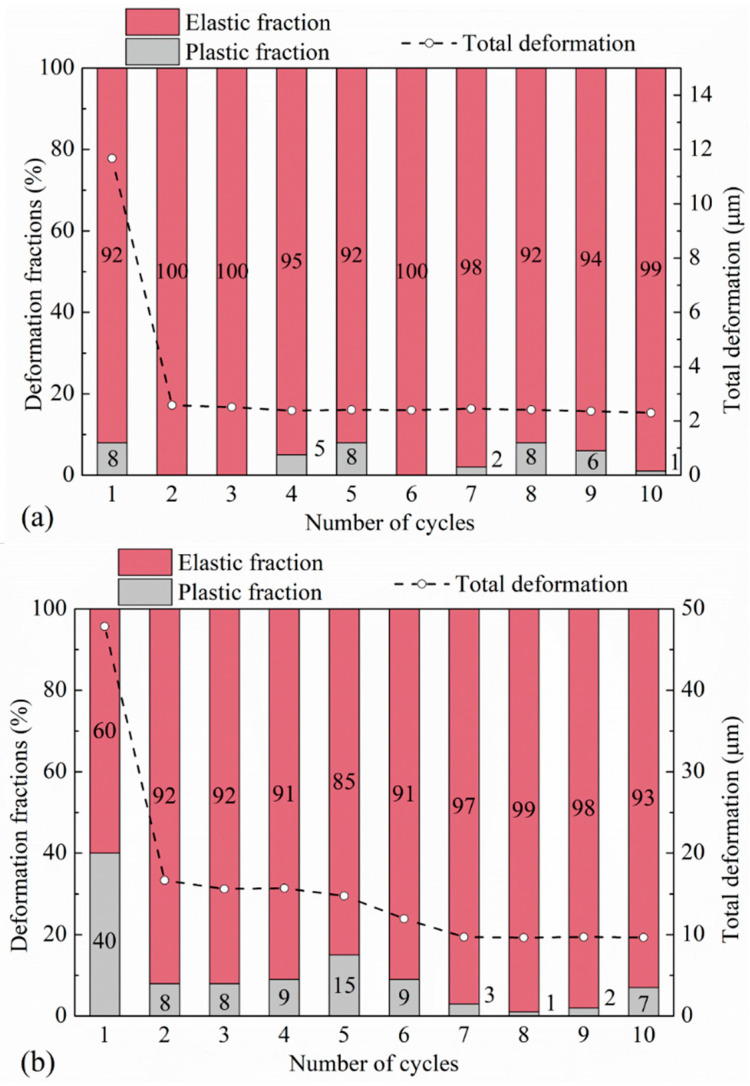
Displacement magnitude and different fractions in preloading for (**a**) LBS–LBS and (**b**) CDG–CDG contacts.

**Figure 13:**
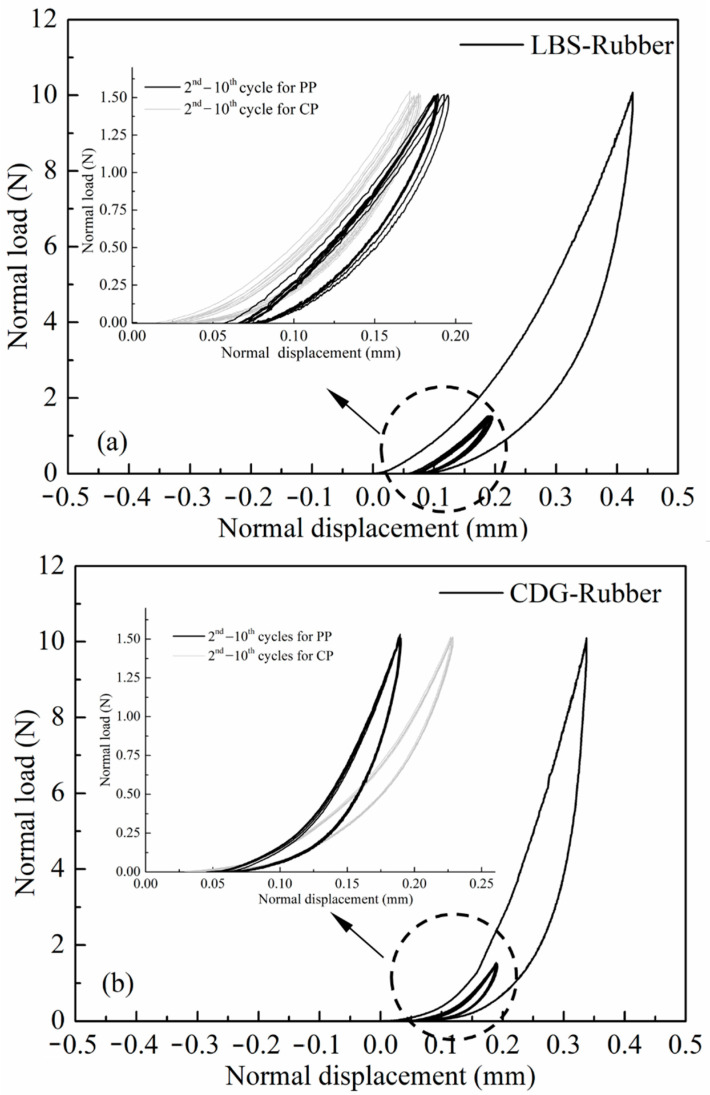
Load–displacement curves of preloading tests for (**a**) LBS–rubber and (**b**) CDG–rubber contacts and their comparisons with cyclic tests (inset Figures).

**Figure 14:**
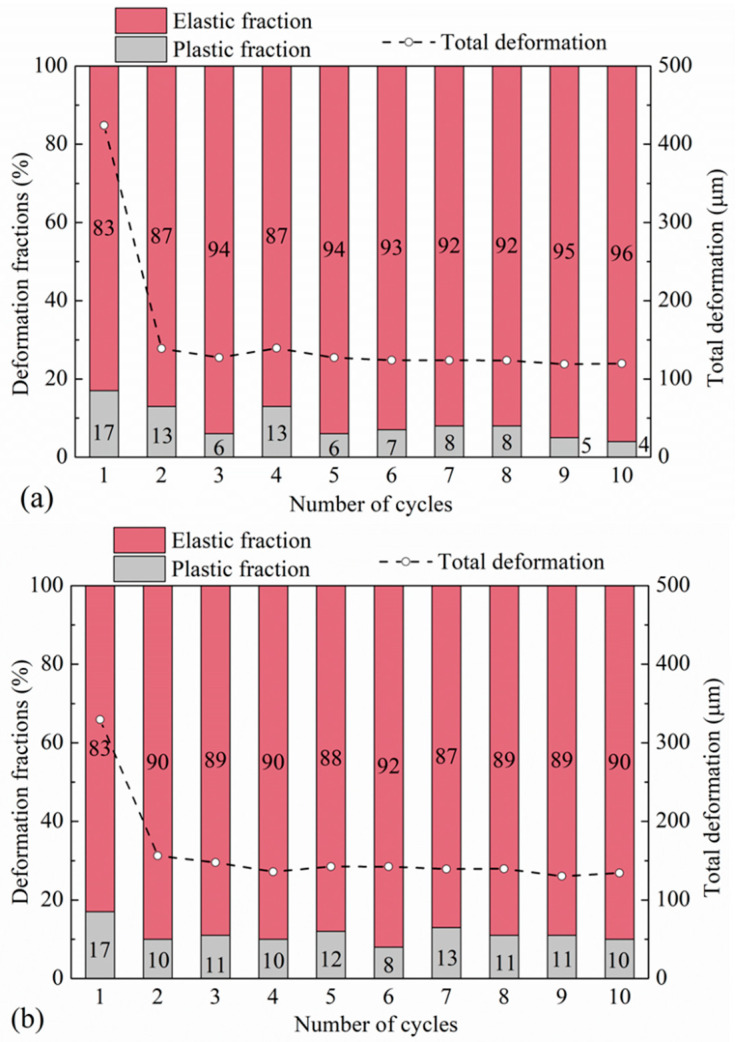
Displacement magnitude and different fractions in preloading tests for (**a**) LBS–rubber and (**b**) CDG–rubber contacts.

The preloading tests were carried out on pure aggregate contacts for LBS and CDG grains (Figure 11 and Figure 12) as well as their composite interfaces with rubber (Figure 13 and Figure 14). In the preloading cycle (cycle-1), where the normal load reached 10 N, a complete hardening behavior was observed for LBS grains, but the CDG grains showed significant brittle damage of the micro-asperities, resulting in fluctuation of the normal load (after reaching approximately 4 N). This behavior is hypothesized to be the result of two major mechanisms: one mechanism is contributed by asperity breakage, which is of a brittle nature, as previous studies would also suggest, on rough interfaces of aggregates or weathered rocks [73,79,93], and a second mechanism is associated with the compression behavior of existing microparticles on the surfaces of the aggregates [77,78]. As discussed in Section 3.1, the CDG grains showed an intermediate behavior of silt- and clay-coated LBS grains (after Kasyap et al. [78]) at 1.5 N normal load. As the normal load further increased, significant particle damage leading to an abrupt drop in the normal load was observed. In the consecutive reloading cycles from cycle-2 to cycle-10 (maximum normal load of 1.5 N), the LBS–LBS and CDG–CDG contacts showed predominantly elastic response, as most parts of the plastic damage occurred in the preloading cycle, even though some small fluctuations of the plastic fractions are acknowledged in Figure 12. A significant decrease (around four times) in the total displacements required to reach 1.5 N normal load for CDG grains was observed when preloading was applied, indicating an increased contact stiffness. Kasyap et al. [78] also observed similar behavior for silt-coated LBS grains owing to the compression of microparticles in the contact region due to excessive normal load in the preloading cycle. From a comparison between CP and PP classes of tests (subsets in Figure 11), a common inference observed for both types of aggregates (LBS and CDG) is that the accumulation of plastic deformations (ratchetting) was higher under the influence of preloading. In the experiments without preloading (CP tests) and with preloading (PP tests), the respective ratchetting displacements were 0.25 and 0.75 μm for LBS grains and 2.5 and 5 μm for CDG grains, respectively. The load–displacement curves of CDG in PP tests also showed an elbow shape during unloading, particularly at very small normal loads below a threshold of 0.25 N, which was not observed in the CP tests on CDG or the experiments on LBS grains. This elbow shape of the unloading curve implies a non-linear response during unloading, which is expected for many material types [94,95], despite the fact that the most important part of the unloading curves in the PP tests can be considered linear. A similar behavior was reported in the recent study by Kasyap et al. [72] based on micro-indentation experiments. Among the different types of geological materials/aggregates examined by [72], elbow-shape curves were observed for recycled concrete aggregate, which has rough surfaces with the presence of microparticles, and this behavior was explained based on the hypothesis of partial relaxation leading to a change of the slope (stiffness) during the unloading process.

#### 3.2.2. Rigid–Soft Interfaces

Similar to the observations in Section 3.1.2 and even though pure LBS and CDG samples had very different responses in the PP tests, LBS–rubber and CDG–rubber specimens subjected to preloading displayed, in general, similar behavior, as shown in Figure 13 and Figure 14. The preloading at 10 N had a limited effect on the subsequent loading cycles for both types of composite interfaces, which displayed (similar to the CP tests) hysteretic behavior and a softer response compared with that of pure aggregate contacts. It is acknowledged, however, by comparing CP (Section 3.1.2) and PP (this section) tests of the composite interfaces, that CDG–rubber samples displayed a slightly stiffer response in PP tests compared with CP tests, whereas the load–displacement curves of LBS–rubber samples displayed a slight shift in the PP tests compared with the respected tests without preloading. Similar to the observations in Figure 12, some fluctuations of the data are observed in Figure 14 in terms of elastic and plastic fractions of displacement, though the absolute values of plastic fractions are not very different between rigid and rigid–soft contacts between cycle-2 and cycle-10 (comparing the data in Figure 12 and Figure 14).

Similar to the discussions in Figure 8 on the behavior of composite interfaces subjected to CP tests, the data on LBS–rubber and CDG–rubber from PP tests in Figure 14 would suggest a dominance of the rubber particle on the behavior of the composite interfaces. Another similarity in the experimental curves in Figure 8 and Figure 13 is that the composite interfaces displayed highly non-linear unloading curves, which is influenced, predominantly, by the viscoelastic behavior of the rubber magnifying relaxation effects. The important influence of the viscoelastic nature of polymeric materials in terms of relaxation (and creep) has been highlighted in the literature for other types of geosynthetics as well [96,97,98].

Macroscopic experiments on sand–rubber mixtures have suggested that the inclusion of rubber prevents particle breakage of the sand, particularly for earth materials of weaker grains [43,44,47,59,91]. Qi et al. [99] attributed the decrease in particle breakage to the absorption of input energy by the rubber particles (through their deformation), which otherwise would have caused sand grain breakage. However, there is a lack of direct experimental evidence at the grain-scale to support this hypothesis, which necessitates micromechanical-based tests to be performed. In the present study, this potentially protective mechanism can be directly examined by calculating the apparent stress at the sand and sand–rubber contacts based on the following expression:

(2) $$\sigma =\frac{F_{N}}{\alpha }$$

where α is the apparent contact area calculated based on Hertz theory, which is assumed to satisfy the following definition (after Johnson [86]):

(3) $$\alpha =\pi \delta R^{*}$$

(4) $$\frac{1}{R^{*}}=\frac{1}{R_{s}}+\frac{1}{R_{r}}$$

where *R_s_* and *R_r_* are the local radii of the sand (rigid) and rubber (soft) particles in the vicinity of their contact, respectively.

In the present study, flat rubber particles were used so that *R_r_* can be considered as infinity, thus:

(5) $$R^{*}=R_{s}$$

**Figure 15:**
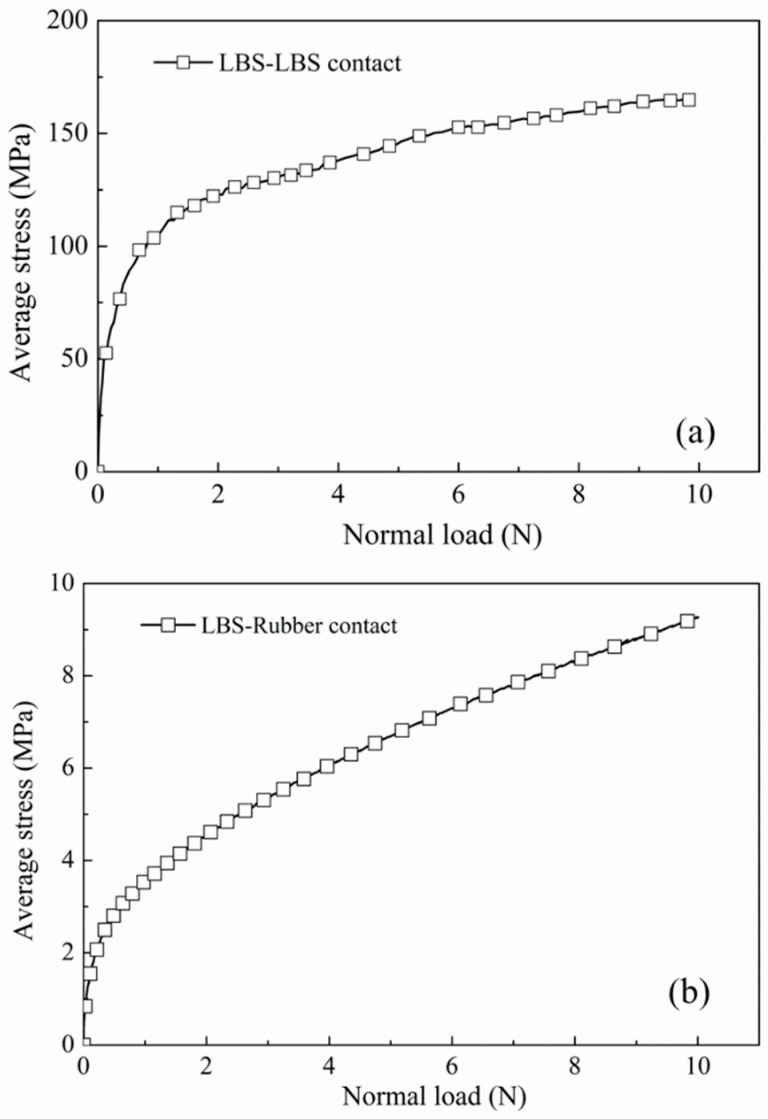
The variation of apparent stress against applied normal load for (**a**) LBS–LBS contact and (**b**) LBS–rubber contact.

LBS–LBS and LBS–rubber interfaces are used as an example due to the simpler calculation of shape parameters and hence the apparent contact stress, as CDG (as well as BLS) particles display high irregularities. Based on the abovementioned expressions, the apparent stress for the virgin cycles of LBS–LBS and LBS–rubber samples in the preloading tests are compared in Figure 15, whose values, due to the influence of local radius and meso-scale morphology of the grains, represent average stresses.

These data suggest that for a normal load of 10 N, the apparent average stress at the contacts of sand grains reaches values of 150 MPa and beyond that. However, for rigid–soft interfaces, the apparent stress is less than 10% (i.e., less than 10 MPa) of that computed for the rigid interfaces, as the soft particles of rubber contribute to the formation of much larger contact area through their deformation, which redistributes (and decreases) the contact stresses. These theoretical (and generally approximate) values are in agreement with the interpretations from the numerical study by Zhang et al. [67] and could provide some quantitative evidence of the interpretations/discussions by Liu et al. [91] that stress concentration is significantly reduced in binary materials such as sand–rubber, which may significantly contribute to the prevention of sand grain breakage.

### 3.3. Energy Dissipation in Cyclic Tests

**Figure 16:**
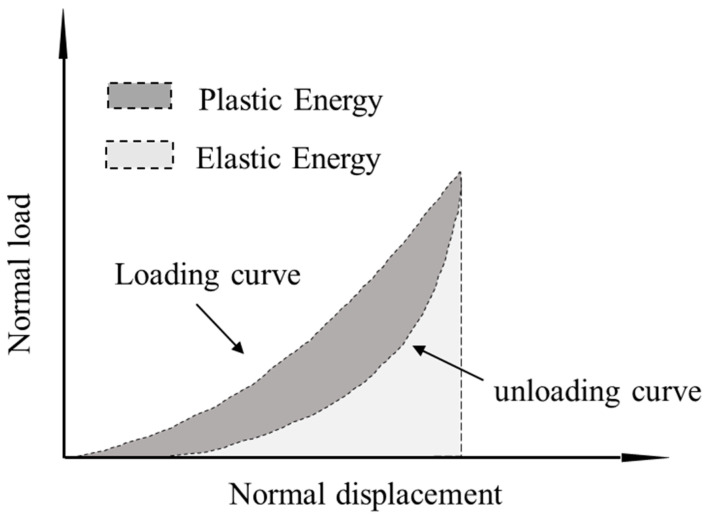
Illustration of elastic and plastic fraction of energy in a complete cycle.

**Figure 17:**
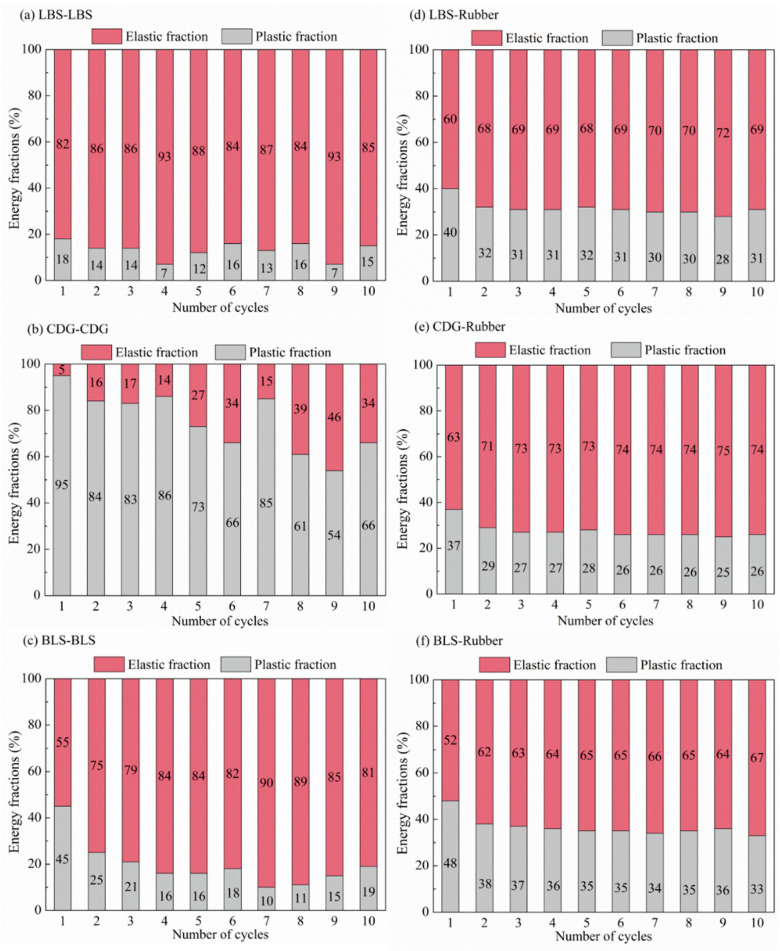
Elastic and plastic fractions of energy input in cyclic tests for LBS–LBS, CDG–CDG, and BLS–BLS (**a**–**c**) and LBS–rubber, CDG–rubber, and BLS–rubber (**d**–**f**).

**Figure 18:**
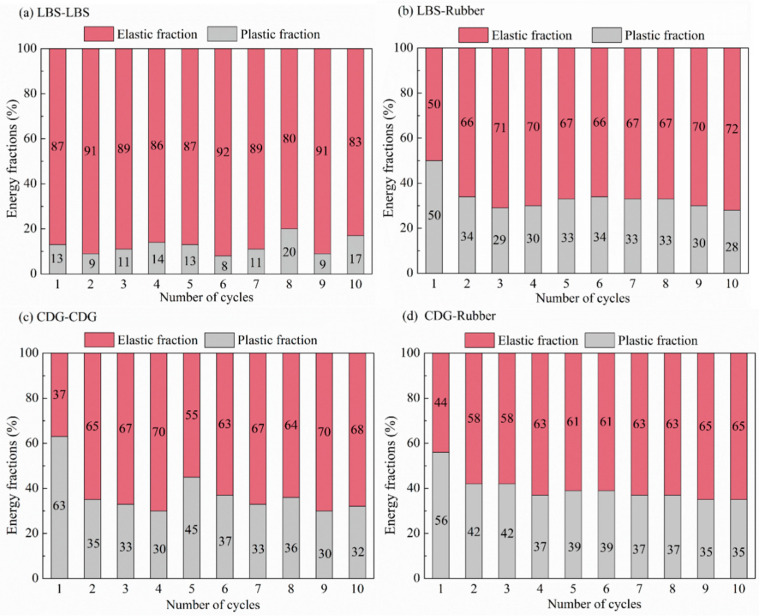
Elastic and plastic fractions of energy input in preloading tests for (**a**) LBS–LBS (**b**)CDG–CDG, (**c**) LBS–rubber, (**d**) and CDG–rubber.

One of the promising applications of granulated and shredded rubber in geotechnical engineering is related to their use as a vibration isolation system due to their high damping (or energy dissipation) capacity. Quantification of the energy dissipation in contact mechanics studies is also important to be obtained for a fundamental understanding of the response of interfaces. In the present study, the energy dissipation of the rigid and rigid–soft interfaces was examined through the estimation of the plastic energy and the elastic and plastic fractions of energy throughout the consecutive loading cycles. An illustrative example of the computation of both elastic and plastic energies is given in Figure 16, where the plastic energy corresponds to the area of the closed-loop in a loading-unloading process, which gives an indication of the dissipated energy, while the elastic energy is defined from the enclosed area below the unloading curve. A summary of the results from both the CP and PP tests on rigid and rigid–soft interfaces is given in Figure 17 and Figure 18.

Based on the data in Figure 17a–c, LBS displayed the lowest values of plastic energy fractions, which is in accordance with the observations in Figure 7. Despite the small fluctuations in the data, some measurable plastic energy fractions are still observed in LBS contacts. Apart from the first loading cycle, where significant plastic energy could be measured for the BLS sample, in the subsequent cycles, the behavior was very much similar to that of LBS (slightly greater plastic energy fractions are acknowledged for BLS contacts compared with those of LBS contacts). For CDG, the behavior was significantly different compared with that of LBS and BLS, with the plastic energy being dominant in all the loading cycles (cycle-1 to cycle-10), demonstrating highly hysteretic behavior. This is in accordance with the observations of the high portion of plastic displacements (Figure 7e), and it is understood that this behavior may have been contributed, predominantly, by the presence of the heavy coating of clay microparticles on CDG surfaces. The roughness of the grains itself should have an influence on the hysteretic behavior observed for all the different types of contacts (LBS, CDG, BLS); however, the significant portion of clay microparticles as the coating is demonstrated to play a key role in the high energy dissipation capacity for CDG grains.

The rigid–soft interfaces displayed a very different behavior, with the viscoelastic rubber providing a homogenization of the response of the different samples (i.e., almost no influence of sand type on the measured elastic and plastic energy fractions) as observed in Figure 17d–f. While for CDG, the inclusion of rubber (by means of sand–rubber interfaces) mitigated the plastic energy fractions compared with pure CDG samples, for both LBS–rubber and BLS–rubber, the plastic fractions were generally significantly increased compared with those of pure LBS and BLS contacts, and similar conclusions could be obtained in the PP tests (Figure 18). However, the data in Figure 18 would also suggest that preloading significantly reduced the plastic energy fractions in CDG, whereas no measurable influence of preloading was observed in LBS.

The data in Figure 17 and Figure 18 suggest that geological materials display some level of hysteretic behavior at their contacts, which is true even for grains with a relatively smooth texture and low roughness, without the presence of natural coating of microparticles (LBS contacts). Increased roughness results in amplifying the hysteretic behavior, which, however, seems to be mitigated as the number of loading cycles increases (BLS contacts); thus, the presence of natural coating of microparticles may play the most dominant role in the continuous dissipation of energy in the subsequent cycles (CDG contacts). In rigid–soft interfaces, the energy dissipation mechanism is different and is attributed, predominantly, to the viscoelastic nature of the rubber, which, simultaneously, prevents plastic displacements but significantly increases the energy dissipation, especially for geological materials, which are dominated by their small-scale surface roughness. An adverse effect was, however, observed for natural grains with a heavy coating of microparticles, as for CDG–rubber, the dissipated energy reduced compared with that of pure CDG contacts.

### 3.4. Theoretical Analysis Using Hertz Fitting

#### 3.4.1. Background of Model and Sensitivity Analysis

It was attempted to apply the Hertz normal contact model [100] to the experimental curves in order to quantify the equivalent (or contact) Young’s modulus of the pure sand and composite interfaces and derive the modulus of the sand and rubber grains. Because of its simplicity, the Hertz model has been widely used in DEM analyses [101] as well as in the discrete-based simulation of sand–rubber mixtures [63,65,67].

Based on the Hertz model, the load–displacement relationship is given as:

(6) $$F_{N}=\frac{4\sqrt{R^{*}}E^{*}\delta_{n}{}^{1.5}}{3}$$

where R*, E*, and *δ_n_* denote the equivalent radius of the contacting grains, the equivalent (contact) Young’s modulus, and normal displacement of the contact, respectively. The equivalent Young’s modulus E* depends on the elastic moduli and Poisson’s ratios of the two bodies in contact as:

(7) $$\frac{1}{E^{*}}=\frac{1-v_{s}{}^{2}}{E_{s}}+\frac{1-v_{r}{}^{2}}{E_{r}}$$

where *v_s_*, *E_s_* and *v_r_*, *E_r_* denote Poisson’s ratio and Young’s modulus of sand and rubber particles, respectively. 

**Figure 19:**
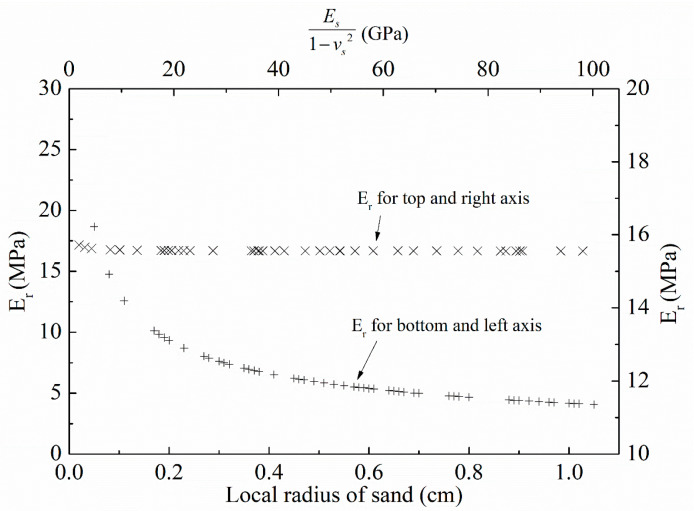
Sensitivity analysis of Hertz model in the estimation of the Young’s modulus of rubber (*E_r_*).

Hertz fitting was applied to LBS–LBS, CDG–CDG, LBS–rubber, and CDG–rubber contacts (in CP tests), but it was avoided for BLS and its composite interface with rubber because of the sharp conical shape of the crushed blue sand in the vicinity of the rigid and rigid–soft interfaces. The subsequent analysis provides a direct fitting of the Hertz model to the experimental curves; thus, model parameter E* is directly estimated from this analysis. This means that in order to estimate *E_r_*, the model parameters of the aggregate (*E_s_* and *v_s_*) must be predefined (estimated from the respected tests on LBS–LBS and CDG–CDG interfaces). However, as these parameters may vary significantly for the natural grains, a sensitivity analysis (with hypothesized values) was performed in order to assess the tolerance of the Hertz model for the composite interfaces (i.e., how sensitive is the estimated *E_r_* value based on the predetermined model parameter values of the sand grains’ rigid body). The analysis using the Hertz model may also be sensitive to changes of the local radius (representing the local radius of the aggregate in this study) as previous studies would suggest [71], so that the sensitivity analysis was further expanded to understand, quantitatively, this influence of the local radius on *E_r_* values, and the results adopting an iteration process are displayed in Figure 19.

These data suggest that even though the back-calculated Young’s modulus of rubber is practically not dependent on the combination of model parameters of the aggregate (*E_s_* and *v_s_*), *E_r_* values are very sensitive to the local radius of the aggregate. Thus, in the subsequent analysis and implementation of the Hertz model, local radii of the sand grains are used. These local radii are determined based on images taken from two microscope digital cameras placed orthogonally, which capture the local shape in the vicinity of the grain contacts (similar to previous studies by [79,81,102]) and the computation of the arithmetic mean of the local radius of the LBS and CDG grains (for both rigid and rigid–soft interfaces).

#### 3.4.2. Data Analysis

**Figure 20:**
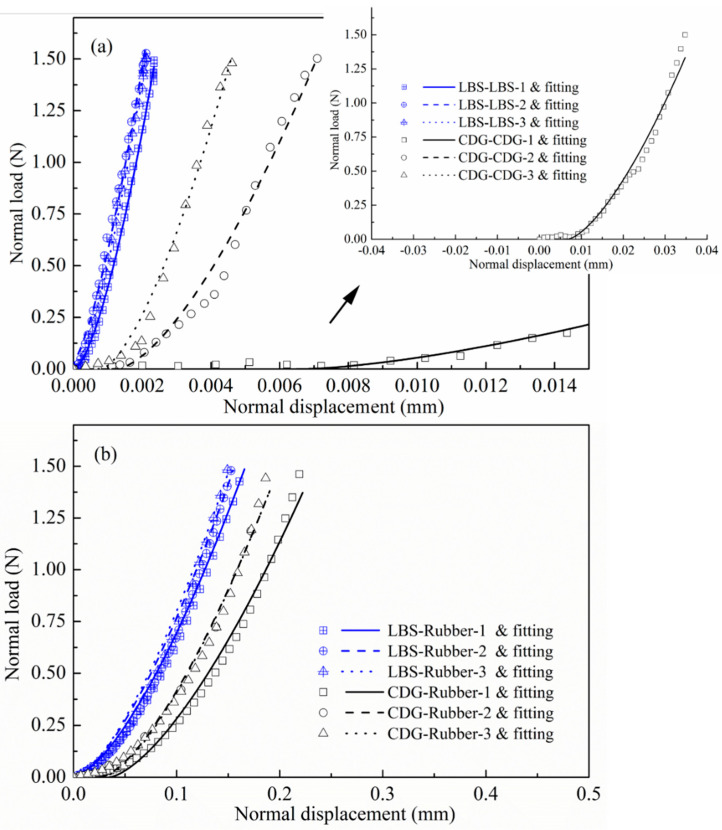
Hertz fitting for the first three cycles of normal loading tests for (**a**) sand–sand contact and (**b**) sand–rubber contact.

Hertz fitting was applied on the first three cycles of the CP tests for rigid and rigid–soft interfaces, and the results (for the loading phase) are summarized in Figure 20 and Table 2. The number displayed next to the sample code expresses the consecutive cycle; for example, “LBS–LBS-2” means data of LBS interfaces (and respected fitting) in cycle-2. From Figure 20 and Table 2, it is observed that for the LBS–LBS sample during the loading process, the resultant *Es* values increased from 53 to 61 GPa from cycle-1 to cycle-3. *E_s_* value corresponding to cycle-1 is in agreement with the reported data by Sandeep and Senetakis [70]. However, for CDG–CDG contacts, cycle-1 revealed a very soft response with *E_s_* value to be equal to 0.7 GPa, and Young’s moduli increased significantly in cycle-2 and cycle-3 of loading. The significant change of the Young’s modulus for the CDG sample as the number of cycles increased, based on Hertzian fitting, is attributed to the plastic deformations during the loading process, which was much less pronounced for LBS, though still measurable (Figure 7). This behavior was also observed by Kasyap et al. [77] in cyclic tests of artificially coated LBS grains with clay microparticles.

For the composite interfaces, Es was taken based on average values (for each material type) from the LBS–LBS and CDG–CDG contacts (equal to 58 and 9.2 GPa, respectively). It is expected that the brittle-to-ductile behavior of geological materials at their contacts leads to the development of plastic deformations (and perhaps some small asperity breakage, though this phenomenon is more pronounced for higher normal loads). Thus, the Young’s modulus of the pure aggregate contacts during the first loading cycle represents an apparent value (after [71]) and is influenced, significantly, by material type, surface morphology, and the presence of coating of microparticles. 

For sand–rubber contacts, it is expected that damage of the sand grain may be prevented so that the *E_s_* value to be used in the analysis for composite interfaces may not be represented effectively by the Young’s modulus of the sand grain contacts as revealed from the first loading cycle. Consecutive cycles on pure sand grain contacts result in a continuous increase of *E_s_*, which is more pronounced for the CDG sample, as the data presented in the previous sections would suggest. Thus, in composite interfaces, two competitive mechanisms are hypothesized to control the resultant Young’s modulus; one is that *E_s_* may be higher in magnitude compared with the derived value from the first cycle in pure sand samples, and the second is that in consecutive loading cycles on the sand–rubber sample, there may be expected negligible change of *E_s_*. Based on these hypotheses and considering that the decision of *E_s_* value will have a minute influence on the resultant *E_r_* (Figure 19), it was compromised to use average *E_s_* values for the composite interfaces based on the respective results on pure sand grain contacts (indicated in Table 2, and these values were considered as constant throughout the three consecutive loading cycles in sand–rubber samples. By applying Hertz fitting on sand–rubber interfaces in CP tests, it was found that *E_r_* ranged between 16–18 and 10–12 MPa for LBS–rubber and CDG–rubber interfaces, respectively. Thus, the application of Hertz fitting with the abovementioned assumptions and compromises provided a reasonable estimation of rubber Young’s modulus with small deviations between LBS–rubber and CDG–rubber samples (note that *E_r_* is by definition independent on sand type, but the small deviations are expected because of the assumptions made in the analysis of the data).

The results in Figure 20 and Table 2 show that the Hertz model applies reasonably well for both LBS and CDG contacts and their respective composite interfaces for a maximum load of 1.5 N. It is acknowledged, however, as previous studies have also reported on geological materials [70,102], that the application of Hertz fitting needs to consider the initial plastic displacements (soft behavior) so that the theoretical curves are shifted to slightly larger displacements prior to the implementation of the fitting process. In the abovementioned analysis, for LBS and LBS–rubber interfaces, fitting was applied without any shift of the theoretical curve (i.e., the Hertz model was applied from the regime of initial displacements); however, for CDG and CDG–rubber, a slight shift to larger displacements, of the order of a few microns, was applied to fit the analytical expression to the experimental data.

## 4. Summary, Conclusions, and Recommendations for Future Research

An experimental micromechanical-based study was presented investigating the cyclic normal contact behavior of rigid–soft interfaces composed of natural sand (rigid grain) against recycled rubber (soft grain). Three different soils were examined, including a natural quartz sand (LBS), grains from completely decomposed granite (CDG), and crushed rock (BLS), while recycled rubber consists of an elastomer type of polymer and is derived from wasted automobile tires. The influence of loading history was examined by performing two types of micromechanical tests. One type involved the application of ten cycles of loading–unloading at a maximum normal load of 1.5 N (CP tests), while the second type involved preloading, in which case cycle-1 was applied at 10 N of normal load, while cycle-2 to cycle-10 were applied at 1.5 N of normal load (PP tests). Elastic and plastic displacements (and their fractions) were defined upon unloading for each cycle. Major conclusions from the study and recommendations for future research are summarized as:

(1) In CP tests of rigid interfaces, LBS grains displayed predominantly elastic response (accounting for only 14% of plastic displacements in cycle-1); however, for CDG, a significant portion of the total displacement was plastic in cycle-1 (80%), and in consecutive cycles, the portion of plastic displacements, despite some fluctuations, stabilized (around 20%). These differences were attributed to the rougher texture and the presence of coating of microparticles on the surfaces of the CDG grains. For BLS, even though their behavior was similar compared to that of LBS in cycle-2 to cycle-10, they displayed greater plastic displacements owing to their rougher texture. A major difference between BLS and CDG was that consecutive cycles resulted in extremely small plastic fractions of displacement for BLS, whereas the cycle number had a continuous influence, with resultant plastic displacement for CDG. These data suggested that the heavy coating of microparticles might be a more prevalent factor controlling the normal contact behavior of the grains.
(2) The data suggested that while for the CP tests, the behavior of the rigid grain contacts was majorly elastic–plastic (i.e., the plasticity of asperities contributes to the measured irreversible strains as well as the irrecoverable compression of the coating), in PP tests, some brittle type of response also contributes to the normal contact behavior of rigid interfaces, especially for CDG.
(3) The composite (rigid–soft) interfaces displayed similar behavior in cycle-2 to cycle-10, which was found almost independent on sand grain type, even though differences were observed in cycle-1. The composite interfaces also displayed some level of plastic deformations, which was subsequently discussed to be an additional mechanism of plastic behavior as observed in element-size experimental tests of sand–rubber mixtures as reported in the literature.
(4) Theoretical analysis of the developed contact stresses in CP tests (maximum normal load of 10 N) revealed that rigid interfaces (using data on LBS) had much greater developed contact stresses of the order of 150 MPa, while the contact stresses were reduced to 10% of these estimated values for rigid–soft interfaces. This theoretical analysis provided a mechanism of mitigation of stress concentration in binary mixtures preventing sand grain breakage.
(5) Quantification of the normal contact stiffness revealed very high values for LBS contacts compared with CDG and BLS, even though all the rigid contacts had at least one order of magnitude higher stiffness compared with that of rigid–soft interfaces. These data may provide some useful guide as input values in DEM simulations of binary (composite) granular materials. The viscoelastic nature of rubber grains also contributed to significantly non-linear behavior in the unloading curves of the composite interfaces, which, as also discussed for other polymeric-based materials in the literature, was attributed to the relaxation of the rubber grains.
(6) The data from the present study comprise a solid basis for future research on composite interfaces. One of these directions can be the investigation of sand–rubber interactions accounting for the potential influence of loading rate (or loading frequency) as well as the influence of temperature on the contact behavior of rigid–soft interfaces. More systematic studies into the behavior of rigid–soft interfaces would also be promising by examining different types of polymeric materials (other than granulated rubber).

## Figures and Tables

**Table 1:** Summary of the testing program and particle geometry parameters.

Type	Loading Path	Local Radius (mm)		No. of Cycles	Maxium Load
		R1	R2		
LBS–LBS	Cyclic Loading	0.24	0.32	10	1.5 N
LBS–rubber		0.72	∞		
CDG–CDG		1.00	0.51		
CDG–rubber		0.99	∞		
BLS–BLS		0.56	0.28		
BLU–rubber		-	∞		
LBS–LBS	Preloading	0.36	0.32	10	10 N(1st cycle)
LBS–rubber		0.81	∞		
CDG–CDG		0.31	0.62		
CDG–rubber		0.47	∞		

**Table 2:** Fitting parameters using Hertz model for the first 3 cycles of loading for different interface types.

Specimen Type	*E_s_* (GPa)	*ν_s_*	*E_r_* (MPa)	*ν_r_*	*E* *	*R^2^*
LBS–LBS-1	53	0.1 *	-	-	27 GPa	0.99
LBS–LBS-2	59		-	-	30 GPa	0.99
LBS–LBS-3	61		-	-	31 GPa	0.99
LBS–rubber-1	58		16	0.5	21 MPa	0.99
LBS–rubber-2	58		18		24 MPa	0.99
LBS–rubber-3	58		18		25 MPa	0.99
CDG–CDG-1	0.7	0.25 **	-	-	0.4 GPa	0.97
CDG–CDG-2	9		-	-	5 GPa	0.98
CDG–CDG-3	18		-	-	10 GPa	0.99
CDG–rubber-1	9.2		10	0.5	13 MPa	0.98
CDG–rubber-2	9.2		12		16 MPa	0.99
CDG–rubber-3	9.2		12		16 MPa	0.99

* *ν_s_* of LBS, after [71] ** *ν_s_* of CDG, after [70].

## Data Availability

Data are available by the corresponding author after reasonable request.
